# 3,3′′-Bis(9-hy­droxy­fluoren-9-yl)-1,1′:3′,1′′-terphen­yl

**DOI:** 10.1107/S1600536813024033

**Published:** 2013-09-07

**Authors:** Konstantinos Skobridis, Vassiliki Theodorou, Georgios Paraskevopoulos, Wilhelm Seichter, Edwin Weber

**Affiliations:** a Department of Chemistry, University of Ioannina, GR-451 10 Ioannina, Greece; b Institut für Organische Chemie, TU Bergakademie Freiberg, Leipziger Strasse 29, D-09596 Freiberg/Sachsen, Germany

## Abstract

The asymmetric unit of the title compound, C_44_H_30_O_2_, contains two independent mol­ecules in which the terminal rings of the terphenyl element are inclined at angles of 36.3 (1) and 22.5 (1)° with respect to the central ring and the dihedral angles between the fluorenyl units are 72.3 (1) and 62.8 (1)°. In the crystal, pairs of O—H⋯O hydrogen bonds link the mol­ecules into inversion dimers. The hy­droxy H atoms not involved in these hydrogen bonds form O—H⋯π inter­actions in which the central terphenyl rings act as acceptors. Weak C—H⋯O contacts and π–π [centroid–centroid distance = 4.088 (2) Å] stacking inter­actions also occur. Taking into account directed non-covalent bonding between the molecules, the crystal is constructed of supramolecular strands extending along the *a*-axis direction.

## Related literature

For the preparation of the starting material for the synthesis of the title compound, see: Staab & Binnig (1967 ▶). For background to organic solid-state inclusion chemistry, see: Atwood *et al.* (1991 ▶). For the design strategy of host compounds, see: Desiraju (1996 ▶). For diol host inclusion complexes, see: Toda (1996 ▶). For host compound 2,2′-bis­(9-hy­droxy-9-fluoren­yl)biphenyl, see: Weber *et al.* (1993 ▶); Skobridis, Paraskevopoulos *et al.* (2011 ▶); Skobridis, Theodorou *et al.* (2011 ▶). For weak O—H⋯π and C—H⋯O inter­actions, see: Desiraju & Steiner (1999 ▶). For π–π stacking inter­actions, see: James (2004 ▶).
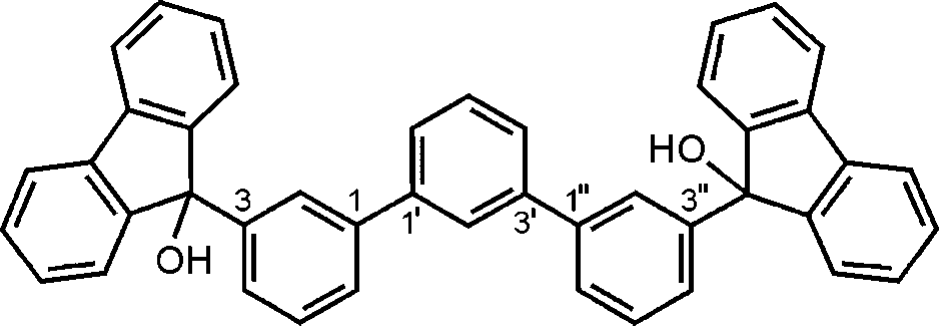


## Experimental

### 

#### Crystal data

C_44_H_30_O_2_
*M* *_r_* = 590.68
Triclinic, 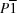

*a* = 11.2292 (3) Å
*b* = 12.4823 (3) Å
*c* = 24.4440 (5) Å
α = 76.070 (1)°
β = 78.080 (1)°
γ = 66.917 (1)°
*V* = 3034.99 (13) Å^3^
*Z* = 4
Mo *K*α radiation
μ = 0.08 mm^−1^
*T* = 150 K
0.32 × 0.18 × 0.06 mm

#### Data collection

Bruker X8 APEXII CCD diffractometer
55787 measured reflections
13774 independent reflections
9930 reflections with *I* > 2σ(*I*)
*R*_int_ = 0.035

#### Refinement

*R*[*F* ^2^ > 2σ(*F* ^2^)] = 0.044
*wR*(*F* ^2^) = 0.109
*S* = 1.03
13774 reflections
845 parameters
4 restraints
H atoms treated by a mixture of independent and constrained refinement
Δρ_max_ = 0.24 e Å^−3^
Δρ_min_ = −0.22 e Å^−3^

### 

Data collection: *APEX2* (Bruker, 2007 ▶); cell refinement: *SAINT* (Bruker, 2007 ▶); data reduction: *SAINT*; program(s) used to solve structure: *SHELXS97* (Sheldrick, 2008 ▶); program(s) used to refine structure: *SHELXL97* (Sheldrick, 2008 ▶); molecular graphics: *ORTEP-3 for Windows* (Farrugia, 2012 ▶); software used to prepare material for publication: *SHELXTL* (Sheldrick, 2008 ▶).

## Supplementary Material

Crystal structure: contains datablock(s) I, New_Global_Publ_Block. DOI: 10.1107/S1600536813024033/rk2410sup1.cif

Structure factors: contains datablock(s) I. DOI: 10.1107/S1600536813024033/rk2410Isup2.hkl

Supplementary material file. DOI: 10.1107/S1600536813024033/rk2410Isup3.cml

Additional supplementary materials:  crystallographic information; 3D view; checkCIF report

## Figures and Tables

**Table 1:** Hydrogen-bond geometry (Å, °) *Cg*1 and *Cg*2 are the centroids of the C20–C25 and C20*A*–C25*A* rings, respectively.

*D*—H⋯*A*	*D*—H	H⋯*A*	*D*⋯*A*	*D*—H⋯*A*
O1—H1⋯O2^i^	0.86 (1)	2.07 (1)	2.894 (2)	163 (1)
O2—H2⋯*Cg*1^ii^	0.84 (1)	3.42 (1)	4.163 (2)	150 (1)
O1*A*—H1*A*⋯O2*A* ^iii^	0.85 (1)	1.99 (1)	2.807 (2)	160 (1)
O2*A*—H2*A*⋯*Cg*2^iv^	0.85 (2)	3.46 (1)	4.169 (2)	145 (1)

Symmetry codes: (i) 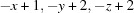; (ii) 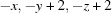; (iii) 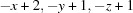; (iv) 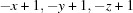.

**Fig. 1.**
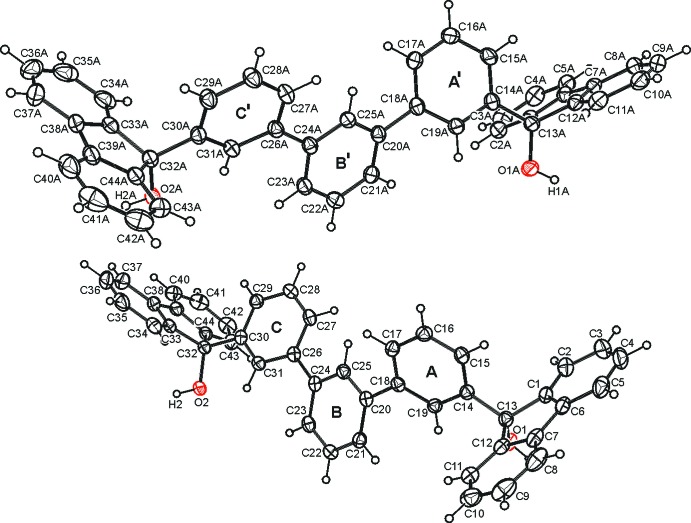
Asymmetric unit of the title compound, showing the atom numbering scheme. Displacement ellipsoids are drawn at the 50% probability level.

**Fig. 2.**
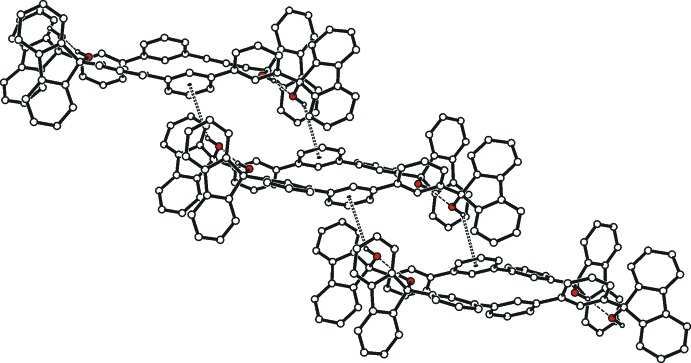
Packing excerpt of the title compound, showing the O—H···O hydrogen-bonded dimers interlinked via O—H···π interactions. O—H···O and O—H···π contacts are presented as broken lines and broken double lines, respectively. Non-relevant hydrogens are omitted for clarity.

**Fig. 3.**
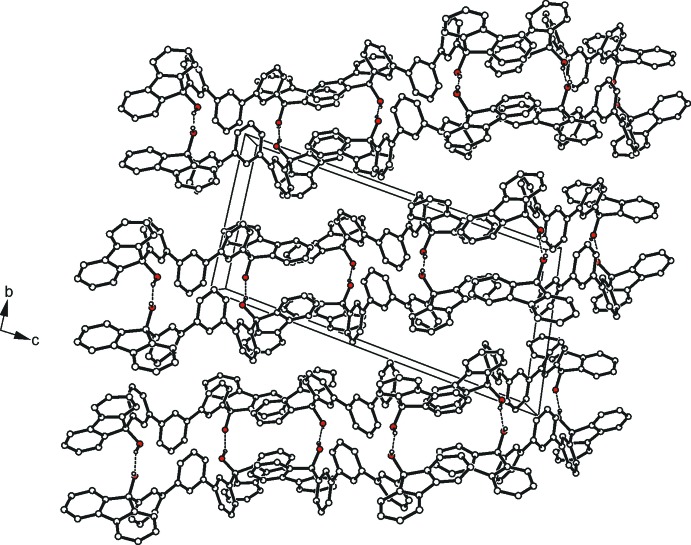
A view along the a-axis of the title compound. Hydrogen-bond type contacts are presented as broken lines. Non-relevant hydrogens are omitted for clarity.

## supplementary crystallographic information

### 1. Comment

In the realm of organic state inclusion chemistry (Atwood *et al.*, 1991), bulkily substituted diols have proven a versatile design strategy for the formation of host compounds (Desiraju, 1996) yielding crystalline host-guest complexes with a variety of guest molecules (Toda, 1996). With regard to that, 2,2'-bis(9-hydroxy-9-fluorenyl)biphenyl is a prototype host structure (Weber *et al.*, 1993; Skobridis, Paraskevopoulos *et al.*, 2010; Skobridis, Theodorou *et al.*, 2011). Following this structural approach, the title compound involving exchange of the central 2,2'-disubstituted biphenyl unit for a 3,3"-disubstituted *m*-terphenyl moiety was prepared and its crystal structure studied unexpectedly showing a solvent-free crystal species on crystallization from ethanol. The compound was found in the space group *P*1 with two crystallographically independent molecules in the asymmetric unit (Fig. 1). The twist angles between the aromatic rings of their terpenyl element are 36.3 (1)° (rings A/B), 22.5 (1)° (rings B/C), 33.8 (1)° (rings A'/B') and 17.2° (rings B'/C') while the mean planes of the fluorenyl units are inclined at angles of 72.3 (1)° and 62.8 (1)°, respectively. The crystal structure (Fig. 2 & Fig. 3) is constructed of inversion dimers which are stabilized by classical O–H···O hydrogen bonds. The hydroxy H atoms excluded from strong hydrogen bonding interlink the molecular dimers *via* O–H···π interactions (Desiraju & Steiner, 1999) with the central rings of the terphenyl units acting as acceptors (O2–H2···C22 = 2.692 (2) Å, 153.3 (1)°; O2A–H2A···C22A = 2.724 (2) Å, 142.5 (1)°). Weak C–H···O contacts (Desiraju & Steiner, 1999) and π–π stacking interactions (James, 2004) (*Cg*(B)···*Cg*(B)^i^, 4.088 (2) Å; symmetry code: (i) 1-*x*, 2-*y*, 2-*z*) complete the pattern of intermolecular interactions.

### 2. Experimental

The starting compound 3,3"-dibromo-1,1':2',1"-terphenyl was prepared according to a literature procedure (Staab & Binnig, 1967). To a stirred solution of this dibromide (2.13 g, 5.5 mmol) in dry diethyl ether (25 ml), *n*-*Bu*Li (1.6 *N* in *n*-hexane, 7.0 ml, 12 mmol) was added dropwise at 195 K under argon. Stirring of the mixture was continued at 253 K for 15 min. Then, fluorenone (1.98 g, 11.0 mmol) in 25 ml of dry diethyl ether was added and the mixture kept at reflux. After completion of the reaction (24 h), which was monitored by *TLC* (hexane/ethyl acetate 2:1, *R_f_* 0.55), the mixture was cooled, quenched with saturated NH_4_Cl solution and extracted with diethyl ether (2 × 30 ml). The combined organic extracts were dried (Na_2_SO_4_) and evaporated. The pale yellow oily residue was precipitated by the addition of hexane and purified by flash chromatography on a SiO_2_ column (hexane/ethyl acetate 2:1) to yield 3.90 g (65 %) of a colourless solid. M.p. = 485-487 K. IR (KBr) 3446, 3037, 1597, 1467, 1448, 1166, 1120, 769, 738. ^1^H NMR (500 MHz, CDCl_3_) δ 2.61 (s, br, 2 H, OH), 7.34-7.62 (m, 21 H, *Ar*H), 7.79-7.92 (m, 7 H, *Ar*H). ^13^C NMR (125 MHz, CDCl_3_) δ 83.7 (CO), 120.2, 124.3, 124.7, 124.8, 126.3, 128.5, 128.7, 129.0, 129.2, 139.7, 141.2, 141.8, 143.8. 150.4 (*Ar*). MS (HR-ESI) m/z: found 613.2126 [M+Na], calc. for C_44_H_30_O_2_ + Na: 613.2138. The melting point (uncorrected) was measured on a hot stage microscope. The IR spectrum was recorded on a Perkin Elmer FT-IR 1600 spectrometer. ^1^H and ^13^C NMR spectra were measured on a Bruker Avance AV-500 spectrometer using (CH_3_)_4_Si as internal standard. The high resolution ESI mass spectrum was obtained using a ThermoFisher Scientific Orbitrap XL spectrometer. Crystals of the title compound suitable for X-ray structural analysis were grown by slow evaporating a solution of the material in ethanol.

### 3. Refinement

The H atoms for hydroxy groups were obtained from the difference electron density map and refined freely. Other aromatic H atoms were positioned geometrically and allowed to ride on their respective parent atoms, with C–H = 0.95Å and *U*_iso_(H) = 1.2*U*_eq_(C).

### Crystal data

**Table:**

C_44_H_30_O_2_	*Z* = 4
*M**_r_* = 590.68	*F*(000) = 1240
Triclinic, *P*1	*D*_x_ = 1.293 Mg m^−^^3^
Hall symbol: -P 1	Melting point = 485–487 K
*a* = 11.2292 (3) Å	Mo *K*α radiation, λ = 0.71073 Å
*b* = 12.4823 (3) Å	Cell parameters from 9885 reflections
*c* = 24.4440 (5) Å	θ = 2.3–28.4°
α = 76.070 (1)°	µ = 0.08 mm^−^^1^
β = 78.080 (1)°	*T* = 150 K
γ = 66.917 (1)°	Irregular, colourless
*V* = 3034.99 (13) Å^3^	0.32 × 0.18 × 0.06 mm

### Data collection

**Table:**

Bruker X8 APEXII CCD diffractometer	9930 reflections with *I* > 2σ(*I*)
Radiation source: fine-focus sealed tube	*R*_int_ = 0.035
Graphite monochromator	θ_max_ = 27.4°, θ_min_ = 0.9°
φ– and ω–scans	*h* = −14→14
55787 measured reflections	*k* = −16→16
13774 independent reflections	*l* = −31→31

### Refinement

**Table:**

Refinement on *F*^2^	Primary atom site location: structure-invariant direct methods
Least-squares matrix: full	Secondary atom site location: difference Fourier map
*R*[*F*^2^ > 2σ(*F*^2^)] = 0.044	Hydrogen site location: inferred from neighbouring sites
*wR*(*F*^2^) = 0.109	H atoms treated by a mixture of independent and constrained refinement
*S* = 1.03	*w* = 1/[σ^2^(*F*_o_^2^) + (0.0457*P*)^2^ + 0.734*P*] where *P* = (*F*_o_^2^ + 2*F*_c_^2^)/3
13774 reflections	(Δ/σ)_max_ < 0.001
845 parameters	Δρ_max_ = 0.24 e Å^−^^3^
4 restraints	Δρ_min_ = −0.22 e Å^−^^3^

### Special details

**Table:**

Geometry. All s.u.'s (except the s.u. in the dihedral angle between two l.s. planes) are estimated using the full covariance matrix. The cell s.u.'s are taken into account individually in the estimation of s.u.'s in distances, angles and torsion angles; correlations between s.u.'s in cell parameters are only used when they are defined by crystal symmetry. An approximate (isotropic) treatment of cell s.u.'s is used for estimating s.u.'s involving l.s.planes.
Refinement. Refinement of *F*^2^ against ALL reflections. The weighted *R*-factor *wR* and goodness of fit *S* are based on *F*^2^, conventional *R*-factors *R* are based on *F*, with *F* set to zero for negative *F*^2^. The threshold expression of *F*^2^ > σ(*F*^2^) is used only for calculating *R*-factors(gt) *etc*. and is not relevant to the choice of reflections for refinement. *R*-factors based on *F*^2^ are statistically about twice as large as those based on *F*, and *R*-factors based on ALL data will be even larger. The bond lengths of the hydroxy groups (O1–H1, O2–H2, O1A–H1A, O2A–H2A) were restraint to target values of 0.84 (1)°.

### Fractional atomic coordinates and isotropic or equivalent isotropic displacement parameters (Å^2^)

**Table:**

	*x*	*y*	*z*	*U*_iso_*/*U*_eq_	
O1	0.94347 (11)	0.99066 (10)	0.89442 (4)	0.0298 (2)	
H1	0.9865 (18)	1.0357 (15)	0.8911 (8)	0.058 (6)*	
O2	−0.06412 (10)	0.85199 (9)	1.09227 (4)	0.0270 (2)	
H2	−0.1445 (10)	0.8725 (18)	1.0921 (9)	0.058 (6)*	
C1	1.12300 (14)	0.86200 (13)	0.83697 (6)	0.0271 (3)	
C2	1.19755 (16)	0.77159 (15)	0.87461 (8)	0.0379 (4)	
H2AA	1.1594	0.7462	0.9114	0.045*	
C3	1.33026 (18)	0.71841 (17)	0.85737 (10)	0.0499 (5)	
H3	1.3832	0.6555	0.8826	0.060*	
C4	1.38572 (17)	0.75594 (18)	0.80409 (10)	0.0503 (5)	
H4	1.4764	0.7183	0.7932	0.060*	
C5	1.31174 (17)	0.84740 (17)	0.76627 (8)	0.0420 (4)	
H5	1.3508	0.8731	0.7297	0.050*	
C6	1.17897 (15)	0.90096 (14)	0.78287 (7)	0.0299 (3)	
C7	1.07732 (15)	0.99960 (14)	0.75333 (6)	0.0292 (3)	
C8	1.08036 (19)	1.06728 (16)	0.69911 (7)	0.0415 (4)	
H8	1.1594	1.0530	0.6739	0.050*	
C9	0.9664 (2)	1.15561 (17)	0.68269 (7)	0.0459 (5)	
H9	0.9676	1.2022	0.6457	0.055*	
C10	0.85090 (19)	1.17759 (15)	0.71886 (8)	0.0421 (4)	
H10	0.7740	1.2392	0.7066	0.051*	
C11	0.84615 (16)	1.11050 (14)	0.77289 (7)	0.0323 (4)	
H11	0.7666	1.1248	0.7977	0.039*	
C12	0.95974 (15)	1.02239 (13)	0.78973 (6)	0.0255 (3)	
C13	0.97803 (14)	0.93475 (13)	0.84594 (6)	0.0238 (3)	
C14	0.88925 (13)	0.86512 (12)	0.85287 (6)	0.0218 (3)	
C15	0.92373 (14)	0.77027 (13)	0.82483 (6)	0.0247 (3)	
H15	1.0080	0.7427	0.8040	0.030*	
C16	0.83589 (14)	0.71609 (13)	0.82719 (6)	0.0251 (3)	
H16	0.8600	0.6519	0.8077	0.030*	
C17	0.71268 (14)	0.75488 (12)	0.85783 (6)	0.0234 (3)	
H17	0.6525	0.7179	0.8588	0.028*	
C18	0.67726 (13)	0.84809 (12)	0.88717 (5)	0.0210 (3)	
C19	0.76591 (13)	0.90268 (12)	0.88367 (6)	0.0218 (3)	
H19	0.7416	0.9674	0.9028	0.026*	
C20	0.54817 (13)	0.88801 (12)	0.92254 (6)	0.0216 (3)	
C21	0.48126 (14)	1.00700 (13)	0.92661 (6)	0.0243 (3)	
H21	0.5180	1.0644	0.9066	0.029*	
C22	0.36119 (14)	1.04162 (13)	0.95977 (6)	0.0258 (3)	
H22	0.3158	1.1229	0.9620	0.031*	
C23	0.30668 (14)	0.95900 (13)	0.98970 (6)	0.0251 (3)	
H23	0.2240	0.9842	1.0120	0.030*	
C24	0.37232 (13)	0.83886 (13)	0.98735 (6)	0.0223 (3)	
C25	0.49253 (14)	0.80609 (13)	0.95310 (6)	0.0230 (3)	
H25	0.5379	0.7249	0.9506	0.028*	
C26	0.32002 (13)	0.74718 (13)	1.02154 (6)	0.0224 (3)	
C27	0.40254 (14)	0.63031 (13)	1.03636 (6)	0.0252 (3)	
H27	0.4936	0.6085	1.0242	0.030*	
C28	0.35372 (14)	0.54571 (13)	1.06855 (6)	0.0258 (3)	
H28	0.4110	0.4659	1.0772	0.031*	
C29	0.22209 (14)	0.57635 (13)	1.08829 (6)	0.0242 (3)	
H29	0.1893	0.5178	1.1105	0.029*	
C30	0.13789 (13)	0.69292 (12)	1.07553 (6)	0.0215 (3)	
C31	0.18631 (14)	0.77647 (13)	1.04124 (6)	0.0224 (3)	
H31	0.1279	0.8551	1.0308	0.027*	
C32	−0.00592 (13)	0.72451 (12)	1.09871 (6)	0.0225 (3)	
C33	−0.07417 (13)	0.67342 (13)	1.06951 (6)	0.0241 (3)	
C34	−0.08402 (15)	0.69123 (14)	1.01235 (6)	0.0303 (3)	
H34	−0.0481	0.7430	0.9855	0.036*	
C35	−0.14767 (16)	0.63166 (15)	0.99506 (7)	0.0375 (4)	
H35	−0.1549	0.6423	0.9559	0.045*	
C36	−0.20056 (17)	0.55699 (16)	1.03447 (8)	0.0424 (4)	
H36	−0.2440	0.5173	1.0219	0.051*	
C37	−0.19141 (16)	0.53890 (15)	1.09183 (8)	0.0370 (4)	
H37	−0.2283	0.4877	1.1186	0.044*	
C38	−0.12712 (14)	0.59729 (13)	1.10932 (6)	0.0266 (3)	
C39	−0.09714 (14)	0.59233 (13)	1.16595 (6)	0.0259 (3)	
C40	−0.12494 (16)	0.52828 (14)	1.21874 (7)	0.0331 (4)	
H40	−0.1745	0.4796	1.2228	0.040*	
C41	−0.07897 (17)	0.53695 (15)	1.26528 (7)	0.0371 (4)	
H41	−0.0965	0.4931	1.3015	0.044*	
C42	−0.00772 (17)	0.60871 (15)	1.25964 (7)	0.0360 (4)	
H42	0.0231	0.6134	1.2920	0.043*	
C43	0.01929 (15)	0.67407 (14)	1.20693 (6)	0.0293 (3)	
H43	0.0679	0.7235	1.2031	0.035*	
C44	−0.02603 (13)	0.66525 (12)	1.16066 (6)	0.0232 (3)	
O1A	1.41120 (11)	0.32518 (9)	0.59705 (5)	0.0357 (3)	
H1A	1.4660 (16)	0.3507 (17)	0.6037 (8)	0.055 (6)*	
O2A	0.44394 (10)	0.54003 (10)	0.39659 (5)	0.0293 (2)	
H2A	0.3615 (10)	0.5663 (18)	0.3979 (9)	0.068 (7)*	
C1A	1.44187 (15)	0.15094 (13)	0.67427 (6)	0.0257 (3)	
C2A	1.32848 (16)	0.17143 (14)	0.71175 (7)	0.0331 (4)	
H2AB	1.2472	0.2245	0.6997	0.040*	
C3A	1.33615 (18)	0.11247 (16)	0.76755 (7)	0.0417 (4)	
H3A	1.2595	0.1261	0.7943	0.050*	
C4A	1.45487 (19)	0.03382 (17)	0.78450 (7)	0.0443 (4)	
H4A	1.4580	−0.0072	0.8226	0.053*	
C5A	1.56883 (17)	0.01375 (15)	0.74710 (7)	0.0369 (4)	
H5A	1.6499	−0.0396	0.7593	0.044*	
C6A	1.56194 (15)	0.07326 (13)	0.69149 (6)	0.0269 (3)	
C7A	1.66334 (14)	0.07059 (12)	0.64249 (6)	0.0261 (3)	
C8A	1.79803 (15)	0.01418 (14)	0.63803 (7)	0.0332 (4)	
H8A	1.8391	−0.0360	0.6700	0.040*	
C9A	1.87136 (16)	0.03244 (15)	0.58611 (8)	0.0387 (4)	
H9A	1.9636	−0.0055	0.5825	0.046*	
C10A	1.81221 (17)	0.10512 (15)	0.53940 (8)	0.0379 (4)	
H10A	1.8642	0.1163	0.5041	0.045*	
C11A	1.67762 (16)	0.16195 (14)	0.54350 (7)	0.0336 (4)	
H11A	1.6372	0.2126	0.5115	0.040*	
C12A	1.60384 (15)	0.14348 (13)	0.59493 (6)	0.0268 (3)	
C13A	1.45695 (14)	0.19901 (12)	0.61050 (6)	0.0263 (3)	
C14A	1.37525 (14)	0.15922 (13)	0.58288 (6)	0.0250 (3)	
C15A	1.41270 (16)	0.04087 (14)	0.57924 (7)	0.0331 (4)	
H15A	1.4936	−0.0144	0.5909	0.040*	
C16A	1.33264 (16)	0.00314 (14)	0.55870 (7)	0.0358 (4)	
H16A	1.3590	−0.0779	0.5562	0.043*	
C17A	1.21453 (15)	0.08276 (13)	0.54183 (6)	0.0298 (3)	
H17A	1.1602	0.0561	0.5278	0.036*	
C18A	1.17481 (14)	0.20167 (13)	0.54526 (6)	0.0237 (3)	
C19A	1.25665 (14)	0.23819 (13)	0.56608 (6)	0.0244 (3)	
H19A	1.2304	0.3191	0.5688	0.029*	
C20A	1.04741 (14)	0.28872 (12)	0.52785 (6)	0.0229 (3)	
C21A	0.97615 (14)	0.38363 (13)	0.55611 (6)	0.0250 (3)	
H21A	1.0091	0.3934	0.5867	0.030*	
C22A	0.85781 (14)	0.46352 (13)	0.53978 (6)	0.0270 (3)	
H22A	0.8099	0.5281	0.5592	0.032*	
C23A	0.80815 (14)	0.45033 (13)	0.49536 (6)	0.0254 (3)	
H23A	0.7262	0.5056	0.4849	0.030*	
C24A	0.87711 (14)	0.35678 (12)	0.46593 (6)	0.0228 (3)	
C25A	0.99691 (14)	0.27705 (12)	0.48319 (6)	0.0231 (3)	
H25A	1.0454	0.2127	0.4637	0.028*	
C26A	0.82490 (14)	0.34188 (12)	0.41805 (6)	0.0231 (3)	
C27A	0.90496 (14)	0.27128 (13)	0.37860 (6)	0.0271 (3)	
H27A	0.9951	0.2310	0.3823	0.032*	
C28A	0.85477 (15)	0.25912 (14)	0.33404 (6)	0.0305 (3)	
H28A	0.9100	0.2081	0.3085	0.037*	
C29A	0.72513 (15)	0.32066 (13)	0.32662 (6)	0.0286 (3)	
H29A	0.6920	0.3138	0.2954	0.034*	
C30A	0.64318 (14)	0.39281 (13)	0.36500 (6)	0.0239 (3)	
C31A	0.69259 (14)	0.40052 (13)	0.41083 (6)	0.0239 (3)	
H31A	0.6355	0.4466	0.4380	0.029*	
C32A	0.50106 (14)	0.46201 (13)	0.35537 (6)	0.0250 (3)	
C33A	0.42463 (14)	0.38268 (13)	0.35927 (7)	0.0277 (3)	
C34A	0.40304 (16)	0.29852 (14)	0.40431 (8)	0.0376 (4)	
H34A	0.4390	0.2827	0.4386	0.045*	
C35A	0.32739 (18)	0.23747 (16)	0.39831 (10)	0.0502 (5)	
H35A	0.3107	0.1799	0.4290	0.060*	
C36A	0.27647 (19)	0.25950 (18)	0.34843 (11)	0.0563 (6)	
H36A	0.2264	0.2159	0.3450	0.068*	
C37A	0.29705 (17)	0.34435 (17)	0.30308 (9)	0.0482 (5)	
H37A	0.2609	0.3597	0.2689	0.058*	
C38A	0.37171 (15)	0.40635 (14)	0.30880 (7)	0.0317 (4)	
C39A	0.41085 (15)	0.49906 (14)	0.26875 (7)	0.0309 (4)	
C40A	0.38515 (18)	0.55247 (16)	0.21365 (7)	0.0427 (4)	
H40A	0.3326	0.5311	0.1956	0.051*	
C41A	0.4372 (2)	0.63715 (17)	0.18554 (7)	0.0487 (5)	
H41A	0.4207	0.6738	0.1476	0.058*	
C42A	0.51331 (19)	0.66985 (15)	0.21146 (7)	0.0437 (4)	
H42A	0.5478	0.7287	0.1914	0.052*	
C43A	0.53934 (16)	0.61658 (14)	0.26691 (7)	0.0330 (4)	
H43A	0.5918	0.6382	0.2849	0.040*	
C44A	0.48756 (14)	0.53195 (13)	0.29503 (6)	0.0264 (3)	

### Atomic displacement parameters (Å^2^)

**Table:**

	*U*^11^	*U*^22^	*U*^33^	*U*^12^	*U*^13^	*U*^23^
O1	0.0302 (6)	0.0376 (6)	0.0289 (6)	−0.0191 (5)	0.0048 (4)	−0.0144 (5)
O2	0.0185 (5)	0.0238 (6)	0.0369 (6)	−0.0063 (5)	−0.0019 (4)	−0.0056 (4)
C1	0.0208 (7)	0.0281 (8)	0.0360 (8)	−0.0108 (6)	−0.0019 (6)	−0.0102 (6)
C2	0.0293 (9)	0.0366 (9)	0.0495 (10)	−0.0126 (8)	−0.0115 (8)	−0.0043 (8)
C3	0.0309 (10)	0.0397 (11)	0.0808 (15)	−0.0065 (8)	−0.0203 (10)	−0.0131 (10)
C4	0.0204 (9)	0.0529 (12)	0.0846 (15)	−0.0093 (9)	0.0009 (9)	−0.0382 (11)
C5	0.0292 (9)	0.0527 (11)	0.0533 (11)	−0.0201 (9)	0.0110 (8)	−0.0305 (9)
C6	0.0240 (8)	0.0346 (9)	0.0387 (9)	−0.0160 (7)	0.0050 (6)	−0.0184 (7)
C7	0.0325 (9)	0.0355 (9)	0.0278 (8)	−0.0216 (7)	0.0033 (6)	−0.0102 (6)
C8	0.0532 (12)	0.0542 (11)	0.0298 (9)	−0.0374 (10)	0.0058 (8)	−0.0089 (8)
C9	0.0715 (14)	0.0488 (11)	0.0296 (9)	−0.0393 (11)	−0.0130 (9)	0.0066 (8)
C10	0.0546 (12)	0.0328 (9)	0.0449 (10)	−0.0204 (9)	−0.0213 (9)	0.0028 (8)
C11	0.0318 (9)	0.0283 (8)	0.0389 (9)	−0.0133 (7)	−0.0058 (7)	−0.0041 (7)
C12	0.0279 (8)	0.0254 (8)	0.0273 (7)	−0.0146 (7)	−0.0010 (6)	−0.0056 (6)
C13	0.0205 (7)	0.0258 (8)	0.0254 (7)	−0.0088 (6)	0.0002 (6)	−0.0070 (6)
C14	0.0202 (7)	0.0225 (7)	0.0216 (7)	−0.0082 (6)	−0.0021 (5)	−0.0014 (5)
C15	0.0194 (7)	0.0283 (8)	0.0239 (7)	−0.0075 (6)	0.0018 (6)	−0.0059 (6)
C16	0.0263 (8)	0.0244 (8)	0.0250 (7)	−0.0086 (6)	−0.0011 (6)	−0.0075 (6)
C17	0.0228 (7)	0.0256 (8)	0.0229 (7)	−0.0110 (6)	−0.0029 (6)	−0.0023 (6)
C18	0.0191 (7)	0.0223 (7)	0.0187 (6)	−0.0061 (6)	−0.0028 (5)	0.0001 (5)
C19	0.0223 (7)	0.0207 (7)	0.0220 (7)	−0.0081 (6)	−0.0021 (5)	−0.0032 (5)
C20	0.0184 (7)	0.0259 (8)	0.0204 (7)	−0.0077 (6)	−0.0023 (5)	−0.0045 (6)
C21	0.0224 (7)	0.0268 (8)	0.0249 (7)	−0.0113 (6)	−0.0027 (6)	−0.0026 (6)
C22	0.0218 (7)	0.0235 (8)	0.0294 (8)	−0.0047 (6)	−0.0040 (6)	−0.0053 (6)
C23	0.0183 (7)	0.0304 (8)	0.0246 (7)	−0.0067 (6)	−0.0004 (6)	−0.0069 (6)
C24	0.0183 (7)	0.0275 (8)	0.0212 (7)	−0.0084 (6)	−0.0026 (5)	−0.0044 (6)
C25	0.0204 (7)	0.0241 (7)	0.0239 (7)	−0.0074 (6)	−0.0017 (6)	−0.0055 (6)
C26	0.0206 (7)	0.0270 (8)	0.0198 (7)	−0.0084 (6)	−0.0016 (5)	−0.0057 (6)
C27	0.0170 (7)	0.0306 (8)	0.0253 (7)	−0.0064 (6)	−0.0005 (6)	−0.0058 (6)
C28	0.0224 (7)	0.0239 (8)	0.0268 (7)	−0.0037 (6)	−0.0034 (6)	−0.0042 (6)
C29	0.0236 (7)	0.0253 (8)	0.0237 (7)	−0.0103 (6)	−0.0018 (6)	−0.0028 (6)
C30	0.0187 (7)	0.0267 (8)	0.0203 (7)	−0.0089 (6)	−0.0019 (5)	−0.0057 (6)
C31	0.0199 (7)	0.0229 (7)	0.0228 (7)	−0.0057 (6)	−0.0022 (5)	−0.0050 (6)
C32	0.0189 (7)	0.0212 (7)	0.0260 (7)	−0.0061 (6)	−0.0021 (6)	−0.0042 (6)
C33	0.0162 (7)	0.0239 (8)	0.0299 (8)	−0.0034 (6)	−0.0032 (6)	−0.0070 (6)
C34	0.0233 (8)	0.0306 (8)	0.0317 (8)	−0.0020 (7)	−0.0064 (6)	−0.0067 (6)
C35	0.0307 (9)	0.0405 (10)	0.0401 (9)	−0.0013 (8)	−0.0155 (7)	−0.0154 (8)
C36	0.0305 (9)	0.0443 (10)	0.0606 (12)	−0.0096 (8)	−0.0159 (8)	−0.0223 (9)
C37	0.0285 (9)	0.0357 (9)	0.0513 (10)	−0.0148 (7)	−0.0034 (7)	−0.0117 (8)
C38	0.0183 (7)	0.0271 (8)	0.0344 (8)	−0.0071 (6)	−0.0011 (6)	−0.0092 (6)
C39	0.0181 (7)	0.0242 (8)	0.0325 (8)	−0.0058 (6)	0.0026 (6)	−0.0078 (6)
C40	0.0288 (8)	0.0286 (8)	0.0372 (9)	−0.0119 (7)	0.0080 (7)	−0.0050 (7)
C41	0.0394 (10)	0.0331 (9)	0.0283 (8)	−0.0093 (8)	0.0058 (7)	−0.0017 (7)
C42	0.0389 (10)	0.0383 (9)	0.0256 (8)	−0.0078 (8)	−0.0032 (7)	−0.0075 (7)
C43	0.0268 (8)	0.0318 (8)	0.0295 (8)	−0.0100 (7)	−0.0019 (6)	−0.0080 (6)
C44	0.0172 (7)	0.0235 (7)	0.0261 (7)	−0.0052 (6)	0.0005 (6)	−0.0054 (6)
O1A	0.0337 (6)	0.0204 (6)	0.0557 (7)	−0.0077 (5)	−0.0239 (6)	0.0004 (5)
O2A	0.0193 (6)	0.0333 (6)	0.0350 (6)	−0.0042 (5)	−0.0057 (5)	−0.0129 (5)
C1A	0.0265 (8)	0.0222 (7)	0.0322 (8)	−0.0086 (6)	−0.0100 (6)	−0.0068 (6)
C2A	0.0287 (8)	0.0328 (9)	0.0410 (9)	−0.0098 (7)	−0.0058 (7)	−0.0135 (7)
C3A	0.0424 (10)	0.0525 (11)	0.0367 (9)	−0.0228 (9)	0.0034 (8)	−0.0174 (8)
C4A	0.0566 (12)	0.0546 (12)	0.0278 (9)	−0.0275 (10)	−0.0092 (8)	−0.0022 (8)
C5A	0.0402 (10)	0.0379 (10)	0.0345 (9)	−0.0141 (8)	−0.0168 (7)	0.0004 (7)
C6A	0.0282 (8)	0.0253 (8)	0.0313 (8)	−0.0096 (7)	−0.0124 (6)	−0.0047 (6)
C7A	0.0253 (8)	0.0209 (7)	0.0352 (8)	−0.0083 (6)	−0.0105 (6)	−0.0046 (6)
C8A	0.0269 (8)	0.0263 (8)	0.0477 (10)	−0.0070 (7)	−0.0141 (7)	−0.0052 (7)
C9A	0.0253 (8)	0.0328 (9)	0.0621 (12)	−0.0123 (7)	−0.0021 (8)	−0.0159 (8)
C10A	0.0374 (10)	0.0377 (10)	0.0445 (10)	−0.0211 (8)	0.0060 (8)	−0.0145 (8)
C11A	0.0388 (10)	0.0325 (9)	0.0339 (8)	−0.0176 (8)	−0.0076 (7)	−0.0033 (7)
C12A	0.0269 (8)	0.0246 (8)	0.0325 (8)	−0.0103 (6)	−0.0094 (6)	−0.0053 (6)
C13A	0.0249 (8)	0.0204 (7)	0.0338 (8)	−0.0058 (6)	−0.0121 (6)	−0.0023 (6)
C14A	0.0231 (7)	0.0259 (8)	0.0249 (7)	−0.0063 (6)	−0.0086 (6)	−0.0018 (6)
C15A	0.0278 (8)	0.0262 (8)	0.0434 (9)	−0.0007 (7)	−0.0185 (7)	−0.0061 (7)
C16A	0.0342 (9)	0.0250 (8)	0.0504 (10)	−0.0034 (7)	−0.0186 (8)	−0.0118 (7)
C17A	0.0278 (8)	0.0308 (9)	0.0348 (8)	−0.0087 (7)	−0.0120 (7)	−0.0092 (7)
C18A	0.0221 (7)	0.0273 (8)	0.0206 (7)	−0.0072 (6)	−0.0058 (6)	−0.0027 (6)
C19A	0.0255 (8)	0.0231 (7)	0.0243 (7)	−0.0071 (6)	−0.0075 (6)	−0.0025 (6)
C20A	0.0204 (7)	0.0246 (8)	0.0228 (7)	−0.0084 (6)	−0.0051 (6)	0.0000 (6)
C21A	0.0252 (8)	0.0283 (8)	0.0231 (7)	−0.0107 (6)	−0.0053 (6)	−0.0040 (6)
C22A	0.0251 (8)	0.0254 (8)	0.0275 (8)	−0.0063 (6)	−0.0012 (6)	−0.0062 (6)
C23A	0.0202 (7)	0.0250 (8)	0.0283 (7)	−0.0060 (6)	−0.0057 (6)	−0.0014 (6)
C24A	0.0200 (7)	0.0237 (7)	0.0221 (7)	−0.0070 (6)	−0.0033 (5)	−0.0002 (6)
C25A	0.0209 (7)	0.0230 (7)	0.0248 (7)	−0.0063 (6)	−0.0037 (6)	−0.0049 (6)
C26A	0.0213 (7)	0.0223 (7)	0.0235 (7)	−0.0069 (6)	−0.0055 (6)	0.0007 (6)
C27A	0.0199 (7)	0.0291 (8)	0.0277 (8)	−0.0042 (6)	−0.0059 (6)	−0.0024 (6)
C28A	0.0254 (8)	0.0342 (9)	0.0280 (8)	−0.0035 (7)	−0.0036 (6)	−0.0106 (6)
C29A	0.0247 (8)	0.0334 (9)	0.0272 (8)	−0.0062 (7)	−0.0081 (6)	−0.0077 (6)
C30A	0.0208 (7)	0.0246 (8)	0.0257 (7)	−0.0077 (6)	−0.0058 (6)	−0.0017 (6)
C31A	0.0204 (7)	0.0235 (7)	0.0251 (7)	−0.0045 (6)	−0.0046 (6)	−0.0036 (6)
C32A	0.0203 (7)	0.0250 (8)	0.0289 (8)	−0.0048 (6)	−0.0059 (6)	−0.0068 (6)
C33A	0.0165 (7)	0.0232 (8)	0.0397 (9)	−0.0023 (6)	−0.0015 (6)	−0.0092 (6)
C34A	0.0249 (8)	0.0270 (9)	0.0502 (10)	−0.0030 (7)	0.0047 (7)	−0.0061 (7)
C35A	0.0304 (10)	0.0282 (9)	0.0819 (15)	−0.0098 (8)	0.0161 (10)	−0.0128 (9)
C36A	0.0318 (10)	0.0431 (11)	0.1030 (18)	−0.0191 (9)	0.0077 (11)	−0.0333 (12)
C37A	0.0290 (9)	0.0500 (12)	0.0758 (14)	−0.0127 (9)	−0.0101 (9)	−0.0295 (10)
C38A	0.0195 (8)	0.0298 (8)	0.0478 (10)	−0.0044 (7)	−0.0079 (7)	−0.0151 (7)
C39A	0.0239 (8)	0.0311 (8)	0.0364 (8)	−0.0012 (7)	−0.0123 (7)	−0.0114 (7)
C40A	0.0402 (10)	0.0451 (11)	0.0404 (10)	−0.0017 (9)	−0.0201 (8)	−0.0140 (8)
C41A	0.0581 (12)	0.0435 (11)	0.0295 (9)	0.0004 (10)	−0.0147 (8)	−0.0036 (8)
C42A	0.0510 (11)	0.0330 (10)	0.0365 (9)	−0.0087 (9)	−0.0020 (8)	−0.0008 (7)
C43A	0.0337 (9)	0.0296 (9)	0.0355 (9)	−0.0099 (7)	−0.0052 (7)	−0.0073 (7)
C44A	0.0215 (7)	0.0246 (8)	0.0311 (8)	−0.0031 (6)	−0.0082 (6)	−0.0059 (6)

### Geometric parameters (Å, º)

**Table:**

O1—C13	1.4276 (17)	O1A—C13A	1.4276 (18)
O1—H1	0.855 (9)	O1A—H1A	0.852 (9)
O2—C32	1.4458 (17)	O2A—C32A	1.4459 (17)
O2—H2	0.837 (9)	O2A—H2A	0.849 (9)
C1—C2	1.378 (2)	C1A—C2A	1.379 (2)
C1—C6	1.402 (2)	C1A—C6A	1.396 (2)
C1—C13	1.520 (2)	C1A—C13A	1.529 (2)
C2—C3	1.392 (2)	C2A—C3A	1.387 (2)
C2—H2AA	0.9500	C2A—H2AB	0.9500
C3—C4	1.380 (3)	C3A—C4A	1.385 (3)
C3—H3	0.9500	C3A—H3A	0.9500
C4—C5	1.383 (3)	C4A—C5A	1.383 (2)
C4—H4	0.9500	C4A—H4A	0.9500
C5—C6	1.391 (2)	C5A—C6A	1.385 (2)
C5—H5	0.9500	C5A—H5A	0.9500
C6—C7	1.467 (2)	C6A—C7A	1.469 (2)
C7—C8	1.392 (2)	C7A—C8A	1.389 (2)
C7—C12	1.399 (2)	C7A—C12A	1.400 (2)
C8—C9	1.381 (3)	C8A—C9A	1.384 (2)
C8—H8	0.9500	C8A—H8A	0.9500
C9—C10	1.380 (3)	C9A—C10A	1.382 (2)
C9—H9	0.9500	C9A—H9A	0.9500
C10—C11	1.386 (2)	C10A—C11A	1.389 (2)
C10—H10	0.9500	C10A—H10A	0.9500
C11—C12	1.380 (2)	C11A—C12A	1.378 (2)
C11—H11	0.9500	C11A—H11A	0.9500
C12—C13	1.531 (2)	C12A—C13A	1.522 (2)
C13—C14	1.522 (2)	C13A—C14A	1.523 (2)
C14—C19	1.3910 (19)	C14A—C19A	1.384 (2)
C14—C15	1.394 (2)	C14A—C15A	1.388 (2)
C15—C16	1.384 (2)	C15A—C16A	1.384 (2)
C15—H15	0.9500	C15A—H15A	0.9500
C16—C17	1.390 (2)	C16A—C17A	1.383 (2)
C16—H16	0.9500	C16A—H16A	0.9500
C17—C18	1.396 (2)	C17A—C18A	1.391 (2)
C17—H17	0.9500	C17A—H17A	0.9500
C18—C19	1.390 (2)	C18A—C19A	1.395 (2)
C18—C20	1.4900 (18)	C18A—C20A	1.4894 (19)
C19—H19	0.9500	C19A—H19A	0.9500
C20—C25	1.391 (2)	C20A—C25A	1.390 (2)
C20—C21	1.395 (2)	C20A—C21A	1.394 (2)
C21—C22	1.3864 (19)	C21A—C22A	1.381 (2)
C21—H21	0.9500	C21A—H21A	0.9500
C22—C23	1.386 (2)	C22A—C23A	1.388 (2)
C22—H22	0.9500	C22A—H22A	0.9500
C23—C24	1.397 (2)	C23A—C24A	1.395 (2)
C23—H23	0.9500	C23A—H23A	0.9500
C24—C25	1.3984 (19)	C24A—C25A	1.4002 (19)
C24—C26	1.489 (2)	C24A—C26A	1.491 (2)
C25—H25	0.9500	C25A—H25A	0.9500
C26—C27	1.394 (2)	C26A—C27A	1.394 (2)
C26—C31	1.4056 (19)	C26A—C31A	1.4027 (19)
C27—C28	1.382 (2)	C27A—C28A	1.388 (2)
C27—H27	0.9500	C27A—H27A	0.9500
C28—C29	1.384 (2)	C28A—C29A	1.381 (2)
C28—H28	0.9500	C28A—H28A	0.9500
C29—C30	1.390 (2)	C29A—C30A	1.392 (2)
C29—H29	0.9500	C29A—H29A	0.9500
C30—C31	1.3861 (19)	C30A—C31A	1.387 (2)
C30—C32	1.5214 (19)	C30A—C32A	1.5248 (19)
C31—H31	0.9500	C31A—H31A	0.9500
C32—C33	1.527 (2)	C32A—C33A	1.521 (2)
C32—C44	1.5304 (19)	C32A—C44A	1.526 (2)
C33—C34	1.382 (2)	C33A—C34A	1.381 (2)
C33—C38	1.401 (2)	C33A—C38A	1.397 (2)
C34—C35	1.391 (2)	C34A—C35A	1.392 (3)
C34—H34	0.9500	C34A—H34A	0.9500
C35—C36	1.384 (3)	C35A—C36A	1.376 (3)
C35—H35	0.9500	C35A—H35A	0.9500
C36—C37	1.384 (2)	C36A—C37A	1.387 (3)
C36—H36	0.9500	C36A—H36A	0.9500
C37—C38	1.388 (2)	C37A—C38A	1.389 (2)
C37—H37	0.9500	C37A—H37A	0.9500
C38—C39	1.472 (2)	C38A—C39A	1.472 (2)
C39—C40	1.391 (2)	C39A—C40A	1.385 (2)
C39—C44	1.398 (2)	C39A—C44A	1.399 (2)
C40—C41	1.385 (2)	C40A—C41A	1.379 (3)
C40—H40	0.9500	C40A—H40A	0.9500
C41—C42	1.385 (2)	C41A—C42A	1.385 (3)
C41—H41	0.9500	C41A—H41A	0.9500
C42—C43	1.394 (2)	C42A—C43A	1.393 (2)
C42—H42	0.9500	C42A—H42A	0.9500
C43—C44	1.375 (2)	C43A—C44A	1.376 (2)
C43—H43	0.9500	C43A—H43A	0.9500
			
C13—O1—H1	110.5 (14)	C13A—O1A—H1A	110.5 (13)
C32—O2—H2	108.7 (14)	C32A—O2A—H2A	109.0 (15)
C2—C1—C6	121.17 (15)	C2A—C1A—C6A	121.42 (14)
C2—C1—C13	128.26 (14)	C2A—C1A—C13A	127.86 (14)
C6—C1—C13	110.55 (13)	C6A—C1A—C13A	110.61 (13)
C1—C2—C3	118.30 (17)	C1A—C2A—C3A	118.30 (15)
C1—C2—H2AA	120.8	C1A—C2A—H2AB	120.9
C3—C2—H2AA	120.8	C3A—C2A—H2AB	120.9
C4—C3—C2	120.78 (18)	C4A—C3A—C2A	120.38 (16)
C4—C3—H3	119.6	C4A—C3A—H3A	119.8
C2—C3—H3	119.6	C2A—C3A—H3A	119.8
C3—C4—C5	121.25 (17)	C5A—C4A—C3A	121.42 (16)
C3—C4—H4	119.4	C5A—C4A—H4A	119.3
C5—C4—H4	119.4	C3A—C4A—H4A	119.3
C4—C5—C6	118.55 (17)	C4A—C5A—C6A	118.44 (16)
C4—C5—H5	120.7	C4A—C5A—H5A	120.8
C6—C5—H5	120.7	C6A—C5A—H5A	120.8
C5—C6—C1	119.95 (16)	C5A—C6A—C1A	120.03 (15)
C5—C6—C7	131.22 (15)	C5A—C6A—C7A	131.35 (14)
C1—C6—C7	108.82 (13)	C1A—C6A—C7A	108.61 (13)
C8—C7—C12	119.53 (16)	C8A—C7A—C12A	120.10 (14)
C8—C7—C6	131.96 (15)	C8A—C7A—C6A	131.28 (14)
C12—C7—C6	108.52 (13)	C12A—C7A—C6A	108.57 (13)
C9—C8—C7	118.76 (16)	C9A—C8A—C7A	118.76 (15)
C9—C8—H8	120.6	C9A—C8A—H8A	120.6
C7—C8—H8	120.6	C7A—C8A—H8A	120.6
C10—C9—C8	121.35 (16)	C10A—C9A—C8A	120.90 (16)
C10—C9—H9	119.3	C10A—C9A—H9A	119.6
C8—C9—H9	119.3	C8A—C9A—H9A	119.6
C9—C10—C11	120.57 (17)	C9A—C10A—C11A	120.72 (16)
C9—C10—H10	119.7	C9A—C10A—H10A	119.6
C11—C10—H10	119.7	C11A—C10A—H10A	119.6
C12—C11—C10	118.45 (16)	C12A—C11A—C10A	118.74 (15)
C12—C11—H11	120.8	C12A—C11A—H11A	120.6
C10—C11—H11	120.8	C10A—C11A—H11A	120.6
C11—C12—C7	121.34 (14)	C11A—C12A—C7A	120.77 (15)
C11—C12—C13	128.05 (13)	C11A—C12A—C13A	128.44 (14)
C7—C12—C13	110.56 (13)	C7A—C12A—C13A	110.69 (13)
O1—C13—C1	112.25 (12)	O1A—C13A—C12A	112.08 (12)
O1—C13—C14	107.04 (11)	O1A—C13A—C14A	107.06 (11)
C1—C13—C14	114.50 (12)	C12A—C13A—C14A	115.11 (12)
O1—C13—C12	113.36 (12)	O1A—C13A—C1A	113.48 (12)
C1—C13—C12	101.52 (11)	C12A—C13A—C1A	101.39 (11)
C14—C13—C12	108.22 (12)	C14A—C13A—C1A	107.77 (12)
C19—C14—C15	118.63 (13)	C19A—C14A—C15A	119.01 (14)
C19—C14—C13	119.65 (12)	C19A—C14A—C13A	120.41 (13)
C15—C14—C13	121.47 (12)	C15A—C14A—C13A	120.29 (13)
C16—C15—C14	120.32 (13)	C16A—C15A—C14A	120.22 (14)
C16—C15—H15	119.8	C16A—C15A—H15A	119.9
C14—C15—H15	119.8	C14A—C15A—H15A	119.9
C15—C16—C17	120.51 (14)	C17A—C16A—C15A	120.35 (15)
C15—C16—H16	119.7	C17A—C16A—H16A	119.8
C17—C16—H16	119.7	C15A—C16A—H16A	119.8
C16—C17—C18	120.05 (14)	C16A—C17A—C18A	120.43 (14)
C16—C17—H17	120.0	C16A—C17A—H17A	119.8
C18—C17—H17	120.0	C18A—C17A—H17A	119.8
C19—C18—C17	118.71 (13)	C17A—C18A—C19A	118.47 (13)
C19—C18—C20	119.99 (13)	C17A—C18A—C20A	121.33 (13)
C17—C18—C20	121.29 (13)	C19A—C18A—C20A	120.20 (13)
C18—C19—C14	121.76 (13)	C14A—C19A—C18A	121.51 (14)
C18—C19—H19	119.1	C14A—C19A—H19A	119.2
C14—C19—H19	119.1	C18A—C19A—H19A	119.2
C25—C20—C21	118.43 (12)	C25A—C20A—C21A	118.57 (13)
C25—C20—C18	120.20 (12)	C25A—C20A—C18A	120.79 (13)
C21—C20—C18	121.36 (12)	C21A—C20A—C18A	120.64 (13)
C22—C21—C20	120.10 (13)	C22A—C21A—C20A	120.16 (14)
C22—C21—H21	120.0	C22A—C21A—H21A	119.9
C20—C21—H21	120.0	C20A—C21A—H21A	119.9
C23—C22—C21	120.77 (14)	C21A—C22A—C23A	120.65 (14)
C23—C22—H22	119.6	C21A—C22A—H22A	119.7
C21—C22—H22	119.6	C23A—C22A—H22A	119.7
C22—C23—C24	120.54 (13)	C22A—C23A—C24A	120.75 (13)
C22—C23—H23	119.7	C22A—C23A—H23A	119.6
C24—C23—H23	119.7	C24A—C23A—H23A	119.6
C23—C24—C25	117.72 (13)	C23A—C24A—C25A	117.56 (13)
C23—C24—C26	121.86 (12)	C23A—C24A—C26A	121.23 (12)
C25—C24—C26	120.38 (13)	C25A—C24A—C26A	121.21 (13)
C20—C25—C24	122.42 (13)	C20A—C25A—C24A	122.31 (13)
C20—C25—H25	118.8	C20A—C25A—H25A	118.8
C24—C25—H25	118.8	C24A—C25A—H25A	118.8
C27—C26—C31	117.89 (13)	C27A—C26A—C31A	117.68 (13)
C27—C26—C24	121.07 (12)	C27A—C26A—C24A	121.73 (13)
C31—C26—C24	121.02 (12)	C31A—C26A—C24A	120.58 (13)
C28—C27—C26	120.87 (13)	C28A—C27A—C26A	120.92 (13)
C28—C27—H27	119.6	C28A—C27A—H27A	119.5
C26—C27—H27	119.6	C26A—C27A—H27A	119.5
C27—C28—C29	120.51 (13)	C29A—C28A—C27A	120.46 (14)
C27—C28—H28	119.7	C29A—C28A—H28A	119.8
C29—C28—H28	119.7	C27A—C28A—H28A	119.8
C28—C29—C30	119.92 (13)	C28A—C29A—C30A	119.84 (14)
C28—C29—H29	120.0	C28A—C29A—H29A	120.1
C30—C29—H29	120.0	C30A—C29A—H29A	120.1
C31—C30—C29	119.37 (13)	C31A—C30A—C29A	119.39 (13)
C31—C30—C32	121.83 (12)	C31A—C30A—C32A	121.58 (13)
C29—C30—C32	118.79 (12)	C29A—C30A—C32A	119.03 (13)
C30—C31—C26	121.34 (13)	C30A—C31A—C26A	121.59 (13)
C30—C31—H31	119.3	C30A—C31A—H31A	119.2
C26—C31—H31	119.3	C26A—C31A—H31A	119.2
O2—C32—C30	107.55 (11)	O2A—C32A—C33A	111.49 (12)
O2—C32—C33	112.49 (11)	O2A—C32A—C30A	107.26 (11)
C30—C32—C33	111.75 (11)	C33A—C32A—C30A	113.03 (12)
O2—C32—C44	111.42 (11)	O2A—C32A—C44A	110.87 (11)
C30—C32—C44	112.09 (11)	C33A—C32A—C44A	101.93 (12)
C33—C32—C44	101.58 (11)	C30A—C32A—C44A	112.30 (12)
C34—C33—C38	120.92 (14)	C34A—C33A—C38A	120.90 (16)
C34—C33—C32	128.55 (14)	C34A—C33A—C32A	128.75 (15)
C38—C33—C32	110.50 (12)	C38A—C33A—C32A	110.35 (13)
C33—C34—C35	118.52 (15)	C33A—C34A—C35A	118.49 (18)
C33—C34—H34	120.7	C33A—C34A—H34A	120.8
C35—C34—H34	120.7	C35A—C34A—H34A	120.8
C36—C35—C34	120.49 (16)	C36A—C35A—C34A	120.77 (18)
C36—C35—H35	119.8	C36A—C35A—H35A	119.6
C34—C35—H35	119.8	C34A—C35A—H35A	119.6
C35—C36—C37	121.35 (16)	C35A—C36A—C37A	121.13 (18)
C35—C36—H36	119.3	C35A—C36A—H36A	119.4
C37—C36—H36	119.3	C37A—C36A—H36A	119.4
C36—C37—C38	118.46 (16)	C36A—C37A—C38A	118.52 (19)
C36—C37—H37	120.8	C36A—C37A—H37A	120.7
C38—C37—H37	120.8	C38A—C37A—H37A	120.7
C37—C38—C33	120.26 (15)	C37A—C38A—C33A	120.18 (17)
C37—C38—C39	131.01 (14)	C37A—C38A—C39A	130.96 (16)
C33—C38—C39	108.69 (13)	C33A—C38A—C39A	108.86 (14)
C40—C39—C44	120.15 (15)	C40A—C39A—C44A	120.02 (16)
C40—C39—C38	131.16 (15)	C40A—C39A—C38A	131.49 (16)
C44—C39—C38	108.67 (12)	C44A—C39A—C38A	108.48 (13)
C41—C40—C39	118.68 (15)	C41A—C40A—C39A	118.79 (17)
C41—C40—H40	120.7	C41A—C40A—H40A	120.6
C39—C40—H40	120.7	C39A—C40A—H40A	120.6
C40—C41—C42	120.83 (15)	C40A—C41A—C42A	121.30 (16)
C40—C41—H41	119.6	C40A—C41A—H41A	119.3
C42—C41—H41	119.6	C42A—C41A—H41A	119.3
C41—C42—C43	120.74 (16)	C41A—C42A—C43A	120.18 (17)
C41—C42—H42	119.6	C41A—C42A—H42A	119.9
C43—C42—H42	119.6	C43A—C42A—H42A	119.9
C44—C43—C42	118.45 (15)	C44A—C43A—C42A	118.63 (16)
C44—C43—H43	120.8	C44A—C43A—H43A	120.7
C42—C43—H43	120.8	C42A—C43A—H43A	120.7
C43—C44—C39	121.13 (14)	C43A—C44A—C39A	121.08 (14)
C43—C44—C32	128.29 (13)	C43A—C44A—C32A	128.56 (14)
C39—C44—C32	110.54 (13)	C39A—C44A—C32A	110.36 (13)
			
C6—C1—C2—C3	−0.8 (2)	C6A—C1A—C2A—C3A	−0.1 (2)
C13—C1—C2—C3	−178.89 (16)	C13A—C1A—C2A—C3A	175.92 (15)
C1—C2—C3—C4	0.4 (3)	C1A—C2A—C3A—C4A	−1.0 (3)
C2—C3—C4—C5	0.2 (3)	C2A—C3A—C4A—C5A	1.5 (3)
C3—C4—C5—C6	−0.4 (3)	C3A—C4A—C5A—C6A	−0.8 (3)
C4—C5—C6—C1	0.1 (2)	C4A—C5A—C6A—C1A	−0.3 (2)
C4—C5—C6—C7	178.64 (16)	C4A—C5A—C6A—C7A	−179.59 (16)
C2—C1—C6—C5	0.6 (2)	C2A—C1A—C6A—C5A	0.7 (2)
C13—C1—C6—C5	178.96 (14)	C13A—C1A—C6A—C5A	−175.89 (14)
C2—C1—C6—C7	−178.32 (15)	C2A—C1A—C6A—C7A	−179.80 (14)
C13—C1—C6—C7	0.08 (17)	C13A—C1A—C6A—C7A	3.56 (17)
C5—C6—C7—C8	2.8 (3)	C5A—C6A—C7A—C8A	−5.3 (3)
C1—C6—C7—C8	−178.51 (17)	C1A—C6A—C7A—C8A	175.30 (16)
C5—C6—C7—C12	−177.63 (16)	C5A—C6A—C7A—C12A	177.18 (16)
C1—C6—C7—C12	1.08 (17)	C1A—C6A—C7A—C12A	−2.18 (17)
C12—C7—C8—C9	0.0 (2)	C12A—C7A—C8A—C9A	0.6 (2)
C6—C7—C8—C9	179.56 (17)	C6A—C7A—C8A—C9A	−176.65 (15)
C7—C8—C9—C10	0.1 (3)	C7A—C8A—C9A—C10A	−0.1 (2)
C8—C9—C10—C11	−0.4 (3)	C8A—C9A—C10A—C11A	0.2 (3)
C9—C10—C11—C12	0.7 (3)	C9A—C10A—C11A—C12A	−0.7 (2)
C10—C11—C12—C7	−0.6 (2)	C10A—C11A—C12A—C7A	1.2 (2)
C10—C11—C12—C13	−177.73 (15)	C10A—C11A—C12A—C13A	177.29 (15)
C8—C7—C12—C11	0.3 (2)	C8A—C7A—C12A—C11A	−1.1 (2)
C6—C7—C12—C11	−179.35 (14)	C6A—C7A—C12A—C11A	176.70 (14)
C8—C7—C12—C13	177.85 (14)	C8A—C7A—C12A—C13A	−177.87 (13)
C6—C7—C12—C13	−1.80 (17)	C6A—C7A—C12A—C13A	−0.06 (17)
C2—C1—C13—O1	55.8 (2)	C11A—C12A—C13A—O1A	−53.1 (2)
C6—C1—C13—O1	−122.45 (13)	C7A—C12A—C13A—O1A	123.37 (14)
C2—C1—C13—C14	−66.5 (2)	C11A—C12A—C13A—C14A	69.6 (2)
C6—C1—C13—C14	115.25 (14)	C7A—C12A—C13A—C14A	−113.97 (14)
C2—C1—C13—C12	177.18 (16)	C11A—C12A—C13A—C1A	−174.42 (15)
C6—C1—C13—C12	−1.08 (16)	C7A—C12A—C13A—C1A	2.02 (16)
C11—C12—C13—O1	−60.3 (2)	C2A—C1A—C13A—O1A	59.9 (2)
C7—C12—C13—O1	122.35 (13)	C6A—C1A—C13A—O1A	−123.76 (14)
C11—C12—C13—C1	179.10 (15)	C2A—C1A—C13A—C12A	−179.77 (15)
C7—C12—C13—C1	1.75 (16)	C6A—C1A—C13A—C12A	−3.40 (16)
C11—C12—C13—C14	58.26 (19)	C2A—C1A—C13A—C14A	−58.50 (19)
C7—C12—C13—C14	−119.08 (13)	C6A—C1A—C13A—C14A	117.87 (13)
O1—C13—C14—C19	28.10 (17)	O1A—C13A—C14A—C19A	−18.55 (18)
C1—C13—C14—C19	153.19 (13)	C12A—C13A—C14A—C19A	−143.86 (14)
C12—C13—C14—C19	−94.41 (15)	C1A—C13A—C14A—C19A	103.86 (15)
O1—C13—C14—C15	−157.72 (13)	O1A—C13A—C14A—C15A	167.68 (14)
C1—C13—C14—C15	−32.64 (19)	C12A—C13A—C14A—C15A	42.36 (19)
C12—C13—C14—C15	79.77 (16)	C1A—C13A—C14A—C15A	−69.92 (17)
C19—C14—C15—C16	0.8 (2)	C19A—C14A—C15A—C16A	0.5 (2)
C13—C14—C15—C16	−173.41 (13)	C13A—C14A—C15A—C16A	174.32 (15)
C14—C15—C16—C17	−0.5 (2)	C14A—C15A—C16A—C17A	−0.2 (3)
C15—C16—C17—C18	−0.9 (2)	C15A—C16A—C17A—C18A	0.0 (3)
C16—C17—C18—C19	1.8 (2)	C16A—C17A—C18A—C19A	−0.1 (2)
C16—C17—C18—C20	−177.17 (12)	C16A—C17A—C18A—C20A	−179.40 (14)
C17—C18—C19—C14	−1.5 (2)	C15A—C14A—C19A—C18A	−0.5 (2)
C20—C18—C19—C14	177.53 (12)	C13A—C14A—C19A—C18A	−174.35 (13)
C15—C14—C19—C18	0.1 (2)	C17A—C18A—C19A—C14A	0.3 (2)
C13—C14—C19—C18	174.49 (12)	C20A—C18A—C19A—C14A	179.63 (13)
C19—C18—C20—C25	−142.93 (14)	C17A—C18A—C20A—C25A	−34.0 (2)
C17—C18—C20—C25	36.04 (19)	C19A—C18A—C20A—C25A	146.67 (14)
C19—C18—C20—C21	36.10 (19)	C17A—C18A—C20A—C21A	145.90 (14)
C17—C18—C20—C21	−144.94 (14)	C19A—C18A—C20A—C21A	−33.4 (2)
C25—C20—C21—C22	−1.1 (2)	C25A—C20A—C21A—C22A	0.2 (2)
C18—C20—C21—C22	179.90 (13)	C18A—C20A—C21A—C22A	−179.69 (13)
C20—C21—C22—C23	0.7 (2)	C20A—C21A—C22A—C23A	0.2 (2)
C21—C22—C23—C24	0.5 (2)	C21A—C22A—C23A—C24A	−0.6 (2)
C22—C23—C24—C25	−1.2 (2)	C22A—C23A—C24A—C25A	0.5 (2)
C22—C23—C24—C26	176.48 (13)	C22A—C23A—C24A—C26A	−179.85 (13)
C21—C20—C25—C24	0.3 (2)	C21A—C20A—C25A—C24A	−0.3 (2)
C18—C20—C25—C24	179.33 (13)	C18A—C20A—C25A—C24A	179.59 (13)
C23—C24—C25—C20	0.9 (2)	C23A—C24A—C25A—C20A	0.0 (2)
C26—C24—C25—C20	−176.89 (13)	C26A—C24A—C25A—C20A	−179.71 (13)
C23—C24—C26—C27	−155.25 (14)	C23A—C24A—C26A—C27A	161.96 (14)
C25—C24—C26—C27	22.4 (2)	C25A—C24A—C26A—C27A	−18.4 (2)
C23—C24—C26—C31	23.0 (2)	C23A—C24A—C26A—C31A	−17.1 (2)
C25—C24—C26—C31	−159.38 (13)	C25A—C24A—C26A—C31A	162.58 (13)
C31—C26—C27—C28	1.1 (2)	C31A—C26A—C27A—C28A	−0.4 (2)
C24—C26—C27—C28	179.40 (13)	C24A—C26A—C27A—C28A	−179.53 (14)
C26—C27—C28—C29	−2.1 (2)	C26A—C27A—C28A—C29A	2.6 (2)
C27—C28—C29—C30	0.3 (2)	C27A—C28A—C29A—C30A	−1.9 (2)
C28—C29—C30—C31	2.4 (2)	C28A—C29A—C30A—C31A	−1.0 (2)
C28—C29—C30—C32	−178.89 (13)	C28A—C29A—C30A—C32A	178.74 (14)
C29—C30—C31—C26	−3.4 (2)	C29A—C30A—C31A—C26A	3.2 (2)
C32—C30—C31—C26	177.94 (13)	C32A—C30A—C31A—C26A	−176.55 (13)
C27—C26—C31—C30	1.6 (2)	C27A—C26A—C31A—C30A	−2.4 (2)
C24—C26—C31—C30	−176.63 (13)	C24A—C26A—C31A—C30A	176.67 (13)
C31—C30—C32—O2	−15.88 (18)	C31A—C30A—C32A—O2A	6.98 (18)
C29—C30—C32—O2	165.48 (12)	C29A—C30A—C32A—O2A	−172.73 (13)
C31—C30—C32—C33	108.04 (15)	C31A—C30A—C32A—C33A	−116.31 (15)
C29—C30—C32—C33	−70.61 (16)	C29A—C30A—C32A—C33A	63.98 (17)
C31—C30—C32—C44	−138.68 (14)	C31A—C30A—C32A—C44A	129.03 (14)
C29—C30—C32—C44	42.67 (18)	C29A—C30A—C32A—C44A	−50.67 (18)
O2—C32—C33—C34	63.47 (18)	O2A—C32A—C33A—C34A	−62.11 (19)
C30—C32—C33—C34	−57.62 (19)	C30A—C32A—C33A—C34A	58.8 (2)
C44—C32—C33—C34	−177.30 (14)	C44A—C32A—C33A—C34A	179.56 (14)
O2—C32—C33—C38	−118.49 (13)	O2A—C32A—C33A—C38A	116.84 (13)
C30—C32—C33—C38	120.42 (13)	C30A—C32A—C33A—C38A	−122.23 (13)
C44—C32—C33—C38	0.74 (15)	C44A—C32A—C33A—C38A	−1.49 (15)
C38—C33—C34—C35	−0.1 (2)	C38A—C33A—C34A—C35A	0.1 (2)
C32—C33—C34—C35	177.76 (14)	C32A—C33A—C34A—C35A	178.95 (14)
C33—C34—C35—C36	0.5 (2)	C33A—C34A—C35A—C36A	0.7 (2)
C34—C35—C36—C37	−0.3 (3)	C34A—C35A—C36A—C37A	−1.1 (3)
C35—C36—C37—C38	−0.2 (2)	C35A—C36A—C37A—C38A	0.6 (3)
C36—C37—C38—C33	0.6 (2)	C36A—C37A—C38A—C33A	0.2 (2)
C36—C37—C38—C39	−177.10 (15)	C36A—C37A—C38A—C39A	179.37 (16)
C34—C33—C38—C37	−0.4 (2)	C34A—C33A—C38A—C37A	−0.5 (2)
C32—C33—C38—C37	−178.64 (13)	C32A—C33A—C38A—C37A	−179.59 (14)
C34—C33—C38—C39	177.73 (13)	C34A—C33A—C38A—C39A	−179.89 (13)
C32—C33—C38—C39	−0.48 (16)	C32A—C33A—C38A—C39A	1.06 (17)
C37—C38—C39—C40	−0.4 (3)	C37A—C38A—C39A—C40A	0.2 (3)
C33—C38—C39—C40	−178.32 (15)	C33A—C38A—C39A—C40A	179.42 (16)
C37—C38—C39—C44	177.87 (16)	C37A—C38A—C39A—C44A	−179.38 (16)
C33—C38—C39—C44	−0.02 (16)	C33A—C38A—C39A—C44A	−0.12 (17)
C44—C39—C40—C41	−1.1 (2)	C44A—C39A—C40A—C41A	0.5 (2)
C38—C39—C40—C41	177.07 (15)	C38A—C39A—C40A—C41A	−179.00 (16)
C39—C40—C41—C42	0.6 (2)	C39A—C40A—C41A—C42A	−0.5 (3)
C40—C41—C42—C43	0.1 (2)	C40A—C41A—C42A—C43A	0.4 (3)
C41—C42—C43—C44	−0.3 (2)	C41A—C42A—C43A—C44A	−0.3 (2)
C42—C43—C44—C39	−0.2 (2)	C42A—C43A—C44A—C39A	0.3 (2)
C42—C43—C44—C32	−178.00 (14)	C42A—C43A—C44A—C32A	−179.63 (15)
C40—C39—C44—C43	0.9 (2)	C40A—C39A—C44A—C43A	−0.4 (2)
C38—C39—C44—C43	−177.61 (13)	C38A—C39A—C44A—C43A	179.16 (14)
C40—C39—C44—C32	179.03 (13)	C40A—C39A—C44A—C32A	179.53 (14)
C38—C39—C44—C32	0.51 (16)	C38A—C39A—C44A—C32A	−0.87 (17)
O2—C32—C44—C43	−62.81 (19)	O2A—C32A—C44A—C43A	62.61 (19)
C30—C32—C44—C43	57.77 (19)	C33A—C32A—C44A—C43A	−178.62 (15)
C33—C32—C44—C43	177.20 (14)	C30A—C32A—C44A—C43A	−57.4 (2)
O2—C32—C44—C39	119.23 (13)	O2A—C32A—C44A—C39A	−117.36 (14)
C30—C32—C44—C39	−120.18 (13)	C33A—C32A—C44A—C39A	1.41 (15)
C33—C32—C44—C39	−0.75 (15)	C30A—C32A—C44A—C39A	122.66 (14)

### Hydrogen-bond geometry (Å, º)

Cg1 and Cg2 are the centroids of the C20–C25 and C20*A*–C25*A* rings, respectively.

**Table:**

*D*—H···*A*	*D*—H	H···*A*	*D*···*A*	*D*—H···*A*
O1—H1···O2^i^	0.86 (1)	2.07 (1)	2.894 (2)	163 (1)
O2—H2···*Cg*1^ii^	0.84 (1)	3.42 (1)	4.163 (2)	150 (1)
O1*A*—H1*A*···O2*A*^iii^	0.85 (1)	1.99 (1)	2.807 (2)	160 (1)
O2*A*—H2*A*···*Cg*2^iv^	0.85 (2)	3.46 (1)	4.169 (2)	145 (1)

Symmetry codes: (i) −*x*+1, −*y*+2, −*z*+2; (ii) −*x*, −*y*+2, −*z*+2; (iii) −*x*+2, −*y*+1, −*z*+1; (iv) −*x*+1, −*y*+1, −*z*+1.
